# Exploring the Polaron Landscape in Germanium Halide Perovskites: CsGeCl_3_, CsGeBr_3_, and CsGeI_3_

**DOI:** 10.1021/acs.jpclett.5c02516

**Published:** 2025-12-26

**Authors:** Mehmet Baskurt, Julia Wiktor

**Affiliations:** Department of Physics, 11248 Chalmers University of Technology, Gothenburg SE-412 96, Sweden

## Abstract

The unique electronic properties of CsGe*X*_3_ perovskites (*X* = Cl, Br, I) make them promising candidates for nonlinear optical applications. Understanding charge localization is needed to fully understand their physical and electronic behavior. Here, we perform a theoretical investigation of electron and hole polaron formation, and self-trapped exciton binding in CsGe*X*_3_ using hybrid density functional theory. We find that polaron stability decreases from Cl to I. In particular, single-electron polarons form highly favorably in CsGeCl_3_ and CsGeBr_3_, whereas single-hole polarons can only be formed in CsGeCl_3_. Double electron polarons are energetically favorable across the series. In addition, CsGeCl_3_ and CsGeBr_3_ exhibit stable self-trapped exciton configurations. These findings constitute a basis for understanding polaronic effects on the electronic properties of CsGe*X*_3_ perovskites and open up access to their optimization in nonlinear optical applications.

## Graphical abstract

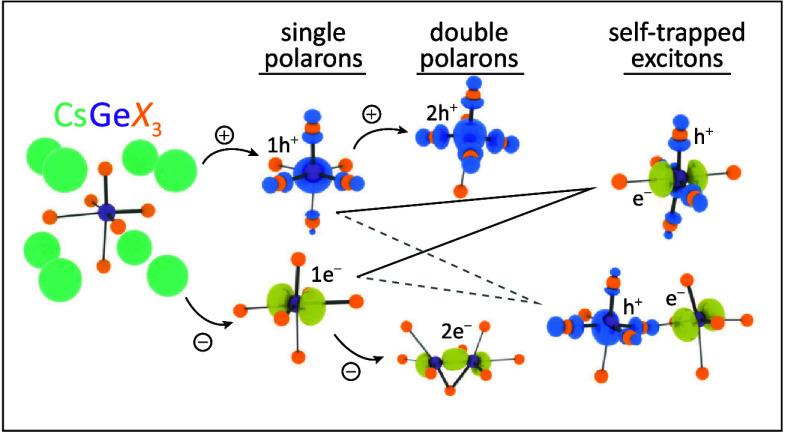

Metal halide perovskites have gained significant interest as promising materials in various applications including solar cells,^1–5^ light-emitting diodes,^6–9^ lasers,^10–12^ and sensors.^13–17^ While most high-performance applications rely on lead-based halide perovskites, concerns about their toxicity have driven interest in lead-free alternatives such as germanium halide perovskites (GHPs), including CsGe*X*_3_ (*X* = Cl, Br, I). GHPs are promising in nonlinear optics applications including lasers, and infrared photodetectors.^18–22^ They exhibit broad-range absorption in the UV–visible spectrum and direct bandgaps tunable over the entire visible range through for example strain engineering, piezoelectric responses, and metal doping.^23–28^

Charge localization can significantly influence electronic and optical properties in perovskites. Localized charge carriers, such as polarons, have been reported to enhance the nonlinear optical response,^29–31^ or reduce charge carrier mobility.^32–35^ In halide double perovskites such as Cs_2_AgBiBr_6_, the emission from self-trapped states has been associated with low-energy peaks in the photoluminescence spectra.^36–39^ Small polaron formation can, in some cases, reduce nonradiative recombination, therefore extending the carrier lifetime and improving device performance.^40,41^ Together, these different effects charge localization can have on materials properties underscore the need to examine polaronic states in emergent lead-free candidates such as CsGe*X*_3_.

In this work, we study excess electrons, holes, and electron–hole pairs in three inorganic GHPs. We show that these materials can host various single and double polaronic states, as well as self-trapped excitons (STEs). Our calculations using nonempirical hybrid-density functionals show a trend in polaron formation energies, with CsGeCl_3_ exhibiting higher stability in comparison to CsGeBr_3_, which in turn has a more stable polaron formation than CsGeI_3_. We report that single electron polarons are favorably formed in both CsGeCl_3_ and CsGeBr_3_ while in CsGeI_3_ they are metastable states. The single hole polaron is only stable in CsGeCl_3_. The double hole polaron (DHP) is stable in CsGeCl_3_, while forms a metastable state with a positive formation energy in CsGeBr_3_ and CsGeI_3_. On the other hand, the double electron polaron (DEP) is stable in all GHPs. Furthermore, we examine the STE binding in these materials, which we report is energetically favorable in CsGeCl_3_ and CsGeBr_3_. Given the localized states we compute, such as single/double polarons and STEs, charge transport is expected to be reduced through carrier localization, and local electron–phonon coupling may contribute to enhanced polarizability and third-order susceptibility, as for example observed in refs 29–32 and 34 , which can enhance optical nonlinearity.

In the present study, we carry out first-principles calculations using the cp2k package^42,43^ to determine the stability and energetics of polaronic states. The cutoff energy for the plane-wave basis set is set to 400 Ry. DZVP-MOLOPT basis sets^44^ are used along with Goedecker-Teter-Hutter pseudopotentials to describe the core–valence interactions.^45^ The auxiliary density matrix method (ADMM) is employed in the calculations,^46^ which are run in the PBE0­(α) hybrid functional level of theory.^47^ The α parameters, 0.32, 0.26, and 0.21 for CsGeCl_3_, CsGeBr_3_, and CsGeI_3_ respectively, are based on ref 48. We validate the applicability of these adapted α values for localized carrier states in the present systems by explicitly testing the Koopmans’ condition for the polaron energy levels (see section 1 in ). In the referenced work, the Koopmans’ condition was verified for a set of halide perovskites by considering intersections of the occupied and unoccupied single-particle levels of a halogen vacancy. This approach has been benchmarked against quasiparticle self-consistent GW (QSGW) across different halide perovskites, including GHPs, and shown good agreement. Although there are alternative methods to compute formation energies of polarons,^49,50^ we consider this approach to provide a reliable and computationally efficient framework for exploring the polaron landscape in CsGe*X*_3_.^51,52^ All calculations are carried out using 320 atom supercells.

The polaron formation energy, *E*_f_, per unit charge is calculated to assess the stability of the self-trapped states as

1 $$E_{f}=\frac{E_{polaron}-E_{pristine}+q\epsilon_{\mathrm{CBM,}\mathrm{VBM}}+E_{corr}}{\left|q\right|}$$

where *E*_polaron_ is the total energy of the charged cell with a polaron, *E*_pristine_ is the total energy of the neutral pristine cell, *q* is the excess charge in the system, ϵ_CBM,VBM_ is the energy of the relevant delocalized state (for *q* = −1, −2 CBM; for *q* = +1, +2 VBM), and *E*_corr_ is the electrostatic finite-size correction term calculated using the Freysoldt-Neugebauer-Van de Walle (FNV) method.^53^ Moreover, we derive finite-size corrections for polaronic single-particle levels using the Falletta-Wiktor-Pasquarello (FWP) method.^54^

We assess the stability of double polarons by their binding energies, $E_{b}^{double}$ , calculated by

2 $$E_{b}^{double}=E_{f}^{double}-E_{f}^{single}$$

where $E_{f}^{double}$ is the formation energy per charge in the double polarons and $E_{f}^{single}$ is the formation energy of the single polaron.

To account for spin–orbit coupling (SOC), we carry out additional calculations using the Vienna *ab initio* Simulation Package (VASP).^55,56^ Within VASP, we run self-consistent field calculations on top of the optimized structures taken from CP2K. We calculate the difference in the polaron formation energy due to SOC to be below 0.08 eV in all cases. Therefore, we neglect it in the following.

To assess the stability of self-trapped excitons, we calculate the binding energy, *E*_b_, as

3 $$E_{b}^{STE}=E_{f}^{STE}-E_{f}^{EP}-E_{f}^{HP}$$

where $E_{f}^{STE}$ is the formation energy of the STE calculated using

4 $$E_{f}^{STE}=E_{STE}-E_{pristine}-\left(\epsilon_{CBM}-\epsilon_{VBM}\right)$$

$E_{f}^{EP}$ is the formation energy of the electron polaron configuration taking part in the STE and $E_{f}^{HP}$ is the formation energy of the hole polaron. Since hole polaron formation is unstable in CsGeBr_3_ and CsGeI_3_, $E_{f}^{HP}$ is considered to be 0 eV in these GHPs.

First, we investigate the ground-state structure of CsGe*X*_3_. We carry out calculations at the PBE0­(α) hybrid functional level to optimize the structures. Our results show that at 0 K GHPs adopt a monoclinic structure. Similar to their room temperature crystal structure, which is reported to be a rhombohedral phase with the *R*3*m* space group,^19,57,58^ the Ge*X*_6_ octahedra exhibit three elongated Ge-*X* bonds, resulting in Ge off-centering as presented in Figure 1. This off-centering introduces spontaneous polarization in the CsGe*X*_3_ perovskites, which can lead to multiple possible polaronic configurations depending on which combination of bonds is deformed. Further information regarding the structural properties of CsGe*X*_3_ is given in the .

**Figure 1:**
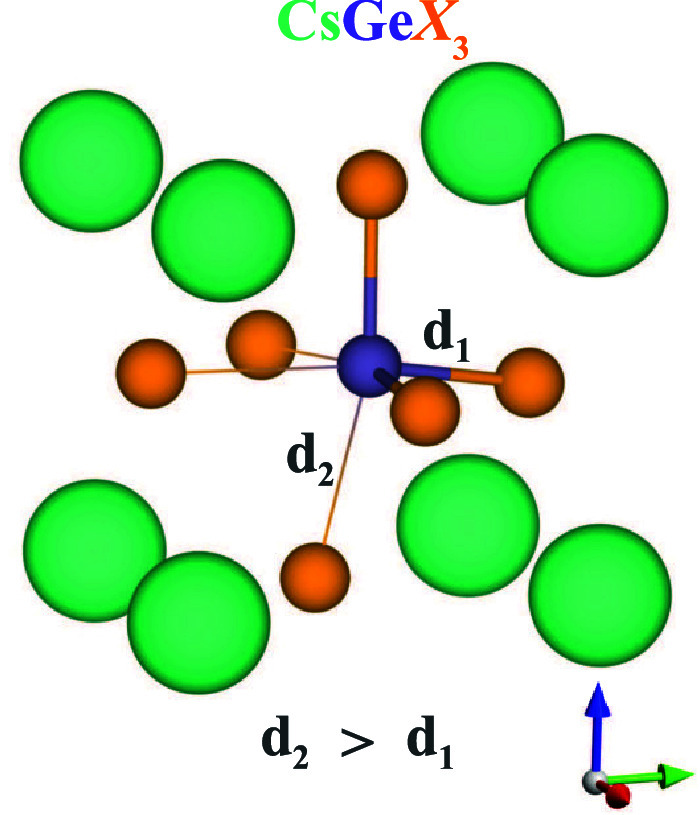
Representation of a single Ge*X*_6_ octahedron. Ge is off-centered within the octahedron.

The lattice parameters of the monoclinic structures of CsGeCl_3_, CsGeBr_3_ (*Cc* space group), and CsGeI_3_ (*Pc* space group) are given in Table 1. Although these space groups have not been previously reported for GHPs, they correspond to the lowest-energy structures within our computational setup, based on 0 K total energy comparisons with other candidate phases. We note that it is particularly important to use the same relaxed ground-state structure for both neutral and charged calculations to avoid uncontrolled structural changes. We use an adiabatic (Born–Oppenheimer) description of polaron formation. For CsGe*X*_3_ perovskites, Fröhlich coupling constants (where we adapt literature values for the effective mass of electron and hole^59,60^ , see SI for more details) show weak-to-intermediate coupling. Our first-principles results nonetheless show stable localized carrier and excitonic states.

**Table 1:** Lattice Parameters of GHPs in the Monoclinic Primitive Cell and Conventional Cell with 320 Atoms

	primitive						conventional cell					
	*a* (Å)	*b* (Å)	*c* (Å)	α (deg)	β (deg)	γ (deg)	*a* (Å)	*b* (Å)	*c* (Å)	α (deg)	β (deg)	γ (deg)
CsGeCl_3_ (*Cc*)	7.72	7.72	7.75	60.34	60.36	60.33	21.94	21.93	21.89	89.58	89.60	90.06
CsGeBr_3_ (*Cc*)	7.99	7.99	8.22	61.02	61.04	60.18	22.97	22.96	22.94	88.29	88.29	88.54
CsGeI_3_ (*Pc*)	8.77	8.46	14.68	90.00	123.98	90.00	24.38	24.37	24.33	87.99	87.95	88.23

Given that the computed polaron formation energies greatly exceed characteristic phonon energies (of the order of tens of meVs), the adiabatic approximation is suitable for describing the self-trapped states, while nonadiabatic electron–phonon effects are expected to primarily renormalize transport and not to alter the presence of self-trapped minima.^61–63^ Therefore, adiabatic descriptions for polaron formation in GHPs are justifiable (see the Supporting Information (SI) for further analysis and discussion on electron–phonon coupling strength). For identifying polaronic states in GHPs, we generate conventional supercells with 320 atoms from the primitive cells. CsGeCl_3_, CsGeBr_3_, and CsGeI_3_ have short Ge-*X* bonds, *d*_1_, with the length of 2.4 Å, 2.6 Å, and 2.8 Å, respectively. The length of the elongated Ge–*X* bonds, *d*_2_, is 3.1 Å in CsGeCl_3_, 3.2 Å in CsGeBr_3_, and 3.4 Å in CsGeI_3_. Among these perovskites, CsGeCl_3_ and CsGeBr_3_ exhibit a three-tilt rotation which is characterized as *a* ^–^ *b* ^–^ *b* ^–^ by Glazer’s notation. On the other hand, monoclinic CsGeI_3_ (Pc space group) exhibits a *a* ^+^ *b* ^–^ *b* ^–^ rotation.

We first identify the possible polaronic configurations in CsGeCl_3_. Due to the anisotropy of the structure, we expect multiple possible configurations of polarons, therefore we consider different initial distortions. We elongate distinct Ge–*X* bond pairs (*d*_2_–*d*_2_, *d*_1_–*d*_2_, and *d*_1_–*d*_1_) to analyze all possible electron polaron configurations. We carry out geometry optimization with a high fraction of exact exchange (α = 0.50) to facilitate charge localization. Here, using a high fraction of exact exchange, we bias the system toward charge localization. This step stabilizes a localized state and lets the lattice to relax into the polaronic configuration. We identify two distinct single electron polaron configurations, which we call EP1 and EP2. The EP1 is a more symmetric structure while the EP2 configuration corresponds to an enhanced off-centering of Ge. We then mimic the initial distortions to form EP1 and EP2 states in CsGeBr_3_ and CsGeI_3_ and optimize the structure with the PBE0(0.50) functional. Resulting systems that contain a localized electron are further optimized with the physically justified, optimal fraction of the exact exchange (CsGeCl_3_: 0.32, CsGeBr_3_: 0.26, CsGeI_3_: 0.21). This two-step procedure ensures that the final polaron properties are evaluated avoiding convergence to artificially delocalized solutions. Charge densities, formation energies, and energy levels are displayed in Figure 2. The formation of a single electron polaron is energetically favorable in CsGeCl_3_ and CsGeBr_3_ with the formation energies of −1.08 eV and −0.23 eV for EP1, −1.05 eV and −0.20 eV for EP2 in CsGeCl_3_, and CsGeBr_3_, respectively. EP1 and EP2 show similarities in their charge density plots and formation energies. However, the major difference between these polaronic configurations lies in the extent of bond distortions and the position of the polaronic single-particle level within the band gap. In CsGeCl_3_, the single-particle level of the EP1 lies 1.87 eV below CBM while that of the EP2 is located at 2.29 eV below CBM, exhibiting a 0.42 eV difference. In CsGeBr_3_, this difference increases to 0.73 eV (for ϵ_CBM_ – ϵ_EP1_ = 0.83 eV, ϵ_CBM_ – ϵ_EP2_ = 1.56 eV). In all cases, EP2 forms a deeper polaronic state. However, as the deformation cost related to this type of electron polaron is higher, the resulting formation energies are similar for EP1 and EP2 in CsGeCl_3_ and CsGeBr_3_.

**Figure 2:**
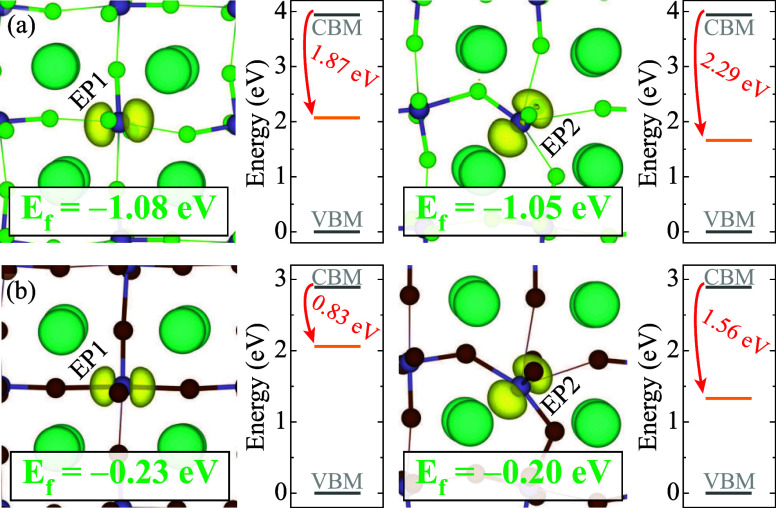
Isodensity surfaces (in yellow) of the single electron polaron configurations and corresponding formation energies for (a) CsGeCl_3_, (b) CsGeBr_3_. Isosurface levels are displayed at 0.005 eV/Å^3^. Zero in the energy scale is set to the VBM.

Next, we assess the stability of a single hole polaron in CsGe*X*_3_. We identify a hole polaron in CsGeCl_3_, referred to as HP, by introducing one additional hole in the system and decreasing Ge–Cl *d*_1_ bond lengths. We find a localized state with a formation energy of only −0.05 eV in CsGeCl_3_, as displayed in Figure 3. After introducing an excess hole and a similar scaled distortion in CsGeBr_3_ and CsGeI_3_, we notice that the extra charge delocalizes. Therefore, we conclude that the formation of the single hole polaron does not occur in these materials.

**Figure 3:**
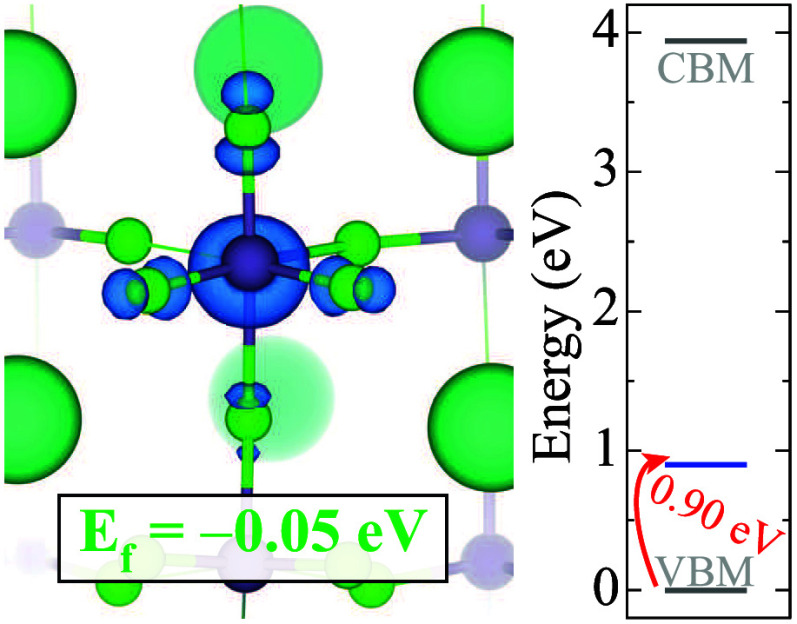
Isodensity surface (in blue) of the single hole polaron and corresponding formation energy for CsGeCl_3_. Isosurface level is displayed at 0.005 eV/Å^3^. Zero in the energy scale is set to the VBM.

In addition to the single electron and hole polarons, we show that double polarons, involving two charges localizing together, can form in CsGe*X*_3_ perovskites. We find that two electrons can localize on two neighboring Ge atoms, while the bridging halogen atom is pushed toward the center of the cage, similar to what has been observed in lead and tin perovskites.^64,65^ We note that we assessed different double polaron configurations for each GHP that differ in Ge pairs as well as the halogen displacement direction. The energy difference between these configurations was found to be negligible, therefore we only report the most stable states. In Figure 4, the stable configuration of the double electron polaron for each CsGe*X*_3_ is shown. We find that double electron polarons can potentially form in all GHPs, with the formation energies of −1.33 eV, −0.60 eV, and −0.20 eV per charge for CsGeCl_3_, CsGeBr_3_, and CsGeI_3_, respectively. By comparing these energies with those found for single electron polarons, we can assess the binding using eq 2. We find binding energies per charge to be −0.25, −0.37, and −0.20 eV for CsGeCl_3_, CsGeBr_3_, and CsGeI_3_, respectively. The negative binding energies imply that double electron polarons can be considered stable. The double electron polarons in GHPs introduce deep states within the band gap with the energy difference between the CBM and the polaronic state of 2.38 eV in CsGeCl_3_, 1.74 eV in CsGeBr_3_, and 1.35 eV in CsGeI_3_.

**Figure 4:**
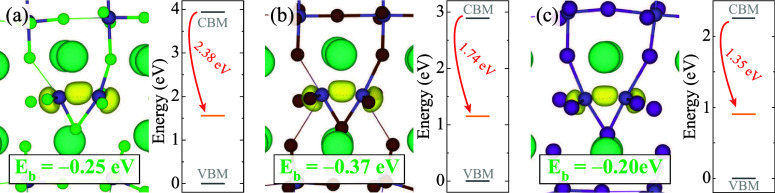
Isodensity surfaces of the double electron polaron configurations and corresponding polaron binding energies of (a) CsGeCl_3_, (b) CsGeBr_3_, (c) CsGeI_3_. Isosurface levels are displayed at 0.005 eV/Å^3^. Zero in the energy scale is set to the VBM.

Next, we explore the formation of double hole polarons in GHPs. We find that two holes can localize on a single Ge site, effectively oxidizing Ge­(II) to Ge­(IV). Without the influence of the stereochemically active 5*s* ^2^ lone-pair, initial octahedral coordination distorts to a tetrahedral arrangement. We analyze distinct double hole polaron configurations that differ by Ge–*X* bonds in the tetrahedral arrangement. The most stable configuration is given in Figure 5. This configuration is only stable in CsGeCl_3_ with the formation energy −0.21 eV per charge (binding energy of −0.16 eV). All stable polaronic configurations across the series are given in Table 2. In CsGeBr_3_ and CsGeI_3_, we only find metastable double hole polaron states, with positive formation energies +0.27 and +0.18 eV per charge, respectively.

**Figure 5:**
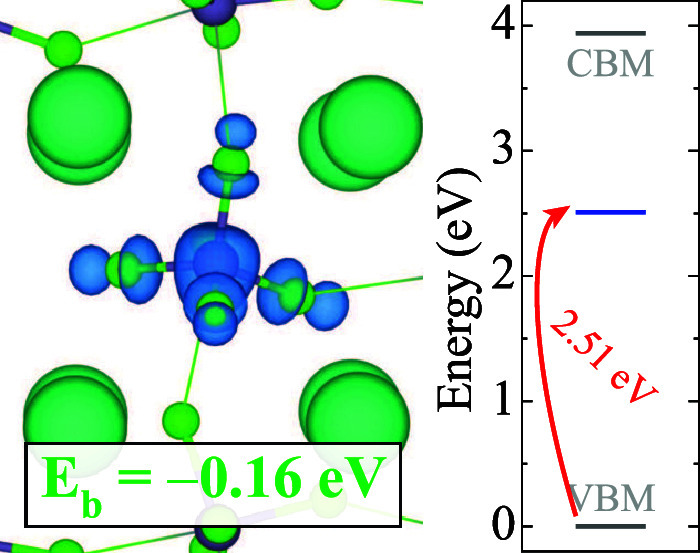
Isosurface of the double hole polaron configuration and corresponding binding energy in CsGeCl_3_. The isosurface levels are displayed at 0.005 eV/Å^3^. Zero in the energy scale is set to the VBM.

**Table 2:** Formation Energy per Charge of Single and Double Polarons, *E*_f_, Energy Difference between the CBM and Polaronic State, *ϵ*_CBM_ – *ϵ*_pol_, and Energy Difference between the Polaronic State and VBM, *ϵ*_pol_ – *ϵ*_VBM_, in CsGe*X*_3_ ^a^

		*E*_f_ (eV/|*q*|)	ϵ_CBM_ – ϵ_pol_ (eV)	ϵ_pol_ – ϵ_VBM_ (eV)
CsGeCl_3_	EP1	–1.08	1.87	2.07
	EP2	–1.05	2.29	1.66
	HP	–0.05	3.04	0.90
	DEP	–1.33	2.38	1.56
	DHP	–0.21	1.43	2.51
				
CsGeBr_3_	EP1	–0.23	0.83	2.06
	EP2	–0.20	1.56	1.33
	DEP	–0.60	1.74	1.15
				
CsGeI_3_	DEP	–0.20	1.35	1.20

a Finite-size corrections are applied to the polaron formation energies and polaronic state *ϵ*_pol_.

Finally, we investigate the formation of self-trapped excitons (STEs), consisting of bound and localized electron–hole pairs in GHPs. We simulate different possible configurations in the triplet state. We find multiple STE configurations in CsGeCl_3_ and CsGeBr_3_, while no stable STE is observed in CsGeI_3_. STE configurations and corresponding formation and binding energies are given in Table 3. In CsGeCl_3_, three distinct stable STE configurations are identified, labeled STE1, STE2, and STE3 (see Figure 6). STE1 and STE2 emerge when an excess hole localizes adjacent to an EP1 or EP2 electron polaron, respectively. These configurations are highly favorable, with formation energies of −1.52 eV and −1.53 eV, and binding energies of −0.39 eV and −0.43 eV for STE1 and for STE2, respectively. Moreover, unlike STE1 and STE2, STE3 involves the localization of both electron and hole on the same Ge atom. This results in a planar arrangement of three Cl atoms surrounding the Ge atom. STE3 forms when the excess hole couples with EP2, with a formation energy of −1.50 eV and a binding energy of −0.40 eV. Moreover, a metastable STE forms when the hole couples with EP1.

**Figure 6:**
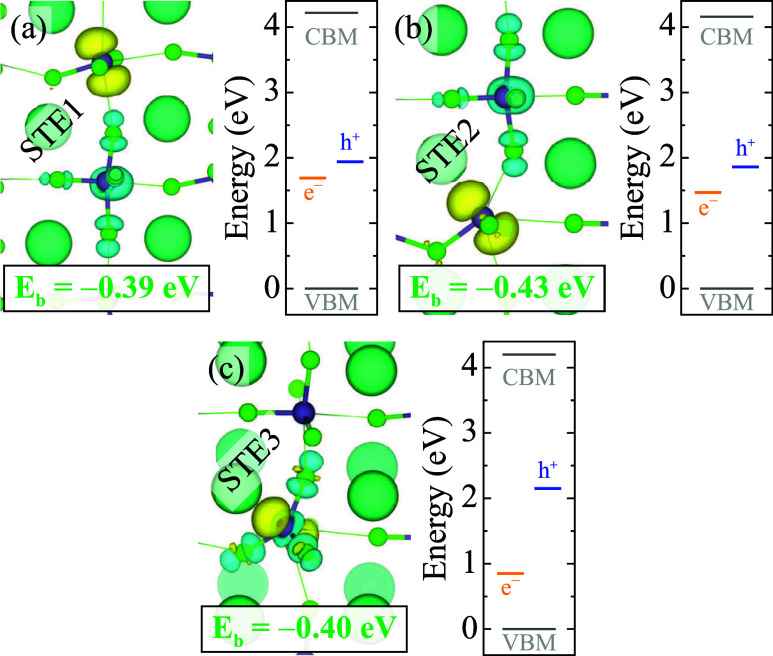
Isosurfaces of the STE configurations in CsGeCl_3_ labeled (a) STE1, (b) STE2, and (c) STE3, with the corresponding energy diagram that shows VBM, CBM, electron (yellow), and hole (blue) levels. Subsets show the binding energy of STEs. The isosurface level is taken at 0.005 eV/Å^3^. Zero in the energy scale is set to the VBM.

**Table 3:** Formation Binding Energies for the STE Configurations in CsGeCl_3_ and CsGeBr_3_

		*E*_f_ (eV)	*E*_b_ (eV)
CsGeCl_3_	STE1	–1.52	–0.39
	STE2	–1.53	–0.43
	STE3	–1.50	–0.40
CsGeBr_3_	STE1	–0.41	–0.18

In CsGeBr_3_, we only find one stable form of STE with electron and hole localized on neighboring Ge sites, resembling STE1. Similar to CsGeCl_3_, EP1 and EP2 configurations can be combined with a hole polaron, leading to the formation of a stable and a metastable STE configuration, respectively. The stable STE, shown in Figure 7, exhibits moderate stability with a formation energy of −0.41 eV and a binding energy of −0.18 eV.

**Figure 7:**
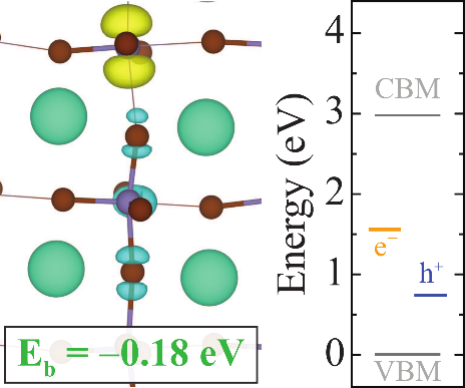
Isosurfaces of the STE configurations in CsGeBr_3_ and the corresponding energy diagram that shows VBM, CBM, electron (yellow), and hole (blue) levels. Subsets show the binding energy of STEs. The isosurface level is taken at 0.005 eV/Å^3^. Zero in the energy scale is set to the VBM.

Information about the metastable STE configurations in CsGeCl_3_ and CsGeBr_3_ is given in the SI. In contrast to CsGeCl_3_ and CsGeBr_3_, no stable or metastable STE configurations were found in CsGeI_3_.

In conclusion, we have studied charge localization in fully inorganic GHPs, namely CsGeCl_3_, CsGeBr_3_, and CsGeI_3_, at the PBE0­(α) hybrid functional level. We found that reduced symmetry in these materials, a consequence of off-centering of Ge, gives rise to multiple, distinct polaronic configurations. We identified two types of single electron polarons, EP1 and EP2. Additionally, stable double electron polarons can form in these GHPs with charges localized on adjacent Ge atoms. Double hole polarons, characterized by local Ge oxidation and a tetrahedral arrangement of Ge–*X* bonds, can also be present in CsGeCl_3_. Notably, a single hole polaron appears only in CsGeCl_3_, whereas the double hole polaron is metastable in CsGeBr_3_ and CsGeI_3_. Furthermore, there are three stable and one metastable STE configurations in CsGeCl_3_, and one stable and one metastable STE configuration in CsGeBr_3_. Since at least one polaronic state is present in all investigated GHPs, accounting for these localized carriers is important when assessing their electronic, optical, and nonlinear optical properties in future studies.

## Supplementary Material





## Data Availability

Simulation data consisting of the final polaron and exciton geometries, and input files are available on ZENODO (doi: 10.5281/zenodo.17129842).

## Notes

Validation of Hartree–Fock exchange fraction in PBE0­(α), finite-size corrections, structural properties of CsGeX_3_, adiabatic treatment of polaron formation in GHPs, orbital character of the polarons, metastable polaron configurations, and metastable self-trapped exciton configurations (PDF)
Transparent Peer Review report available (PDF)
