# Supplementary material for: Exploring the Polaron Landscape in Germanium Halide Perovskites: CsGeCl3, CsGeBr3, and CsGeI3
Source: J Phys Chem Lett. 2025 Dec 26;17(6):1569–75. doi: 10.1021/acs.jpclett.5c02516 (PMC12908147; doi:10.1021/acs.jpclett.5c02516)
Supplement: Supplementary file 1 [file jz5c02516_si_001.pdf]

Supporting Information:

Exploring the Polaron Landscape in Germanium Halide Perovskites: $CsGeCl_3$, $CsGeBr_3$, and $CsGeI_3$

Mehmet Baskurt[1] and Julia Wiktor[1,*]

[1] *Department of Physics, Chalmers University of Technology, SE-41296, Gothenburg, Sweden*
[*] *julia.wiktor@chalmers.se*

1 Fraction of Hartree-Fock exchange in PBE0(α)

In this work, we investigate polaron formation in $CsGeX_3$ perovskites using the PBE0(α) hybrid functional. The fractions of Hartree-Fock exchange (α) are taken from Ref. 1, where α was determined non-empirically by enforcing the generalized Koopmans' condition for halogen vacancy states. To validate the applicability of these α values for localized carrier states in the present systems, we explicitly tested the Koopmans' condition for the polaron energy levels, specifically for the electron polaron configurations EP1 and EP2.

For EP1, we find the optimal value for the HF exchange fraction α to be 0.36, 0.32, and 0.29 for $CsGeCl_3$, $CsGeBr_3$, and $CsGeI_3$, respectively, after applying finite-size corrections to the single-particle levels (see Figure 1). For EP2, the corresponding values are 0.36, 0.30, and 0.27, respectively. These values are higher than those reported for vacancy states in Ref. 1 (0.32, 0.26, 0.21). However, Ref. 1 also found that other localized states, modeled using adjustable potential probes, yielded somewhat higher α values. This indicates that such variations are expected and confirms that the previously reported α values provide a self-interaction-free description of localized charges, which we therefore adopt throughout the main calculations for consistency.

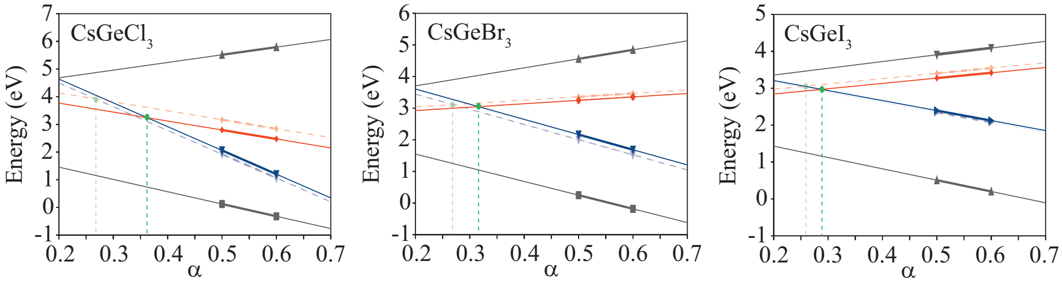

Figure 1: Band edges and single particle levels of electron polaron EP1 in $CsGeCl_3$, $CsGeBr_3$, and $CsGeI_3$. Blue and red lines represent the occupied and unoccupied single-particle levels, respectively. Single-particle levels with solid lines are corrected levels, while dashed lines are the uncorrected levels. Green points indicate the HF fraction that satisfies Koopmans' condition.

2 Finite-size corrections

We apply finite-size corrections to polaron formation energies to increase the accuracy. The electrostatic finite-size correction term, E_{corr}, is derived using the Freysoldt-Neugebauer-Van de Walle (FNV) method,[2] and the finite-size corrections for polaronic single-particle levels, ϵ_{corr}, using the Falletta-Wiktor-Pasquarello (FWP) method.[3] The electrostatic finite-size correction terms are listed in Table S1.

	Single polaron			Double polaron		
	q	E_{corr} (eV)	ϵ_{corr} (eV)	q (eV)	E_{corr} (eV)	ϵ_{corr}
$CsGeCl_3$	+	+0.07	−0.14	+2	+0.28	−0.28
	−	+0.07	+0.14	−2	+0.28	+0.28
$CsGeBr_3$	+	+0.08	−0.16	+2	+0.32	−0.32
	−	+0.08	+0.16	−2	+0.32	+0.32
$CsGeI_3$	+	+0.04	−0.07	+2	+0.15	−0.15
	−	+0.04	+0.07	−2	+0.15	+0.15

Table S1: Finite-size corrections for each computed quantity. Localized charge, q; electrostatic finite-size correction term, E_{corr}; single-particle level finite-size correction term,ϵ_{corr}.

3 Structural properties of CsGe$\boldsymbol{X}_3$

For identifying polaronic states, it is important to use the same relaxed ground-state structure for both neutral and charged calculations to avert uncontrolled changes in the structure. Here, we first investigate the ground-state structure of CsGeX_3 perovskites at PBE0(α) level. We find that the monoclinic Cc ($CsGeCl_3$ and $CsGeBr_3$) and Pc ($CsGeI_3$) phases are the lowest in energy at 0 K. Relative to the previously reported rhombohedral R3m phase, the energy differencese are 0.3 meV/atom, 1.4 meV/atom, 4.4 meV/atom for $CsGeCl_3$, $CsGeBr_3$, and $CsGeI_3$, respectively. Although the energy differences are small, these differences are sufficient to establish the monoclinic ground states. Importantly, since the polaron formation energies depend on the underlying lattice, we consistently use these relaxed ground states for all neutral and charged calculations.

4 Adiabatic treatment of polaron formation in GHPs

In this work, we evaluate polaron formation adiabatically by adding or removing electronic charge and relaxing the structure on a single potential energy surface. Since polarons with small formation energies can be sensitive to non-adiabatic effects, we quantify the long-range electron-phonon coupling in GHPs via the Fröhlich constant, α,

$$\alpha = \frac{e^2}{4\pi\epsilon\hbar}\sqrt{\frac{m^*}{2\hbar\omega_{\text{LO}}}}(\frac{1}{\epsilon_\infty} - \frac{1}{\epsilon_0}), \tag{1}$$

where m^* is the carrier effective mass, ω_{LO} is the polar LO phonon frequency at Γ, ϵ_∞ is the high-frequency dielectric tensor, andϵ_0 is the static dielectric tensor. Values are given in Table S2.

The Fröhlich constants show weak to intermediate coupling strength across the series, with 2.54 ($CsGeCl_3$) > 1.28 ($CsGeI_3$) > 0.63 ($CsGeBr_3$). These values point to intermediate-coupling behavior rather than the strong small-polaron limit.[4,5] In this α range, static relaxations dominate the stabilization energy while non-adiabatic corrections primarily renormalize transport and are not expected to alter the presence of self-trapped minima, therefore not alter polaron formation qualitatively.

	ω_{LO} (THz)	ϵ_∞ (x,y,z)	ϵ_0 (x,y,z)	m_e^*/m_e	m_h^*/m_e	α
$CsGeCl_3$	9.13	3.70, 3.70, 3.70	13.91, 13.91, 12.13	0.27	0.28	2.54
$CsGeBr_3$	6.20	6.55, 6.55, 6.55	11.07, 11.07, 11.07	0.18	0.18	0.63
$CsGeI_3$	5.05	6.22, 6.22, 6.22	20.96, 20.96, 20.96	0.14	0.14	1.28

Table S2: LO phonon frequency at Γ (ω_{LO}), directional components of high-frequency (ϵ_∞) and static (ϵ_0) dielectric tensors, electron (m_e^*/m_e) and hole (m_h^*/m_e) effective masses,[6,7] and resulting Fröhlich constant α.

5 Orbital character of the polarons

To identify the orbital character of the localized charge carriers, we analyzed spin-polarized atom-projected density of states for the Ge atom hosting the polaron in the supercell. In the electron polaron configurations, the in-gap state is dominated by Ge(p) character, whereas the hole polaron level is primarily Ge(s). Moreover, single hole polaron shows a non-negligible Ge(p) contribution. We note that these observations hold for all the three perovskites.

6 Metastable polaron configurations

In addition to the stable polarons discussed in the main text, GHPs can also exhibit metastable polaronic configurations. These configurations refer to localized charge states that correspond to local minima in the potential energy surface, but are higher in energy than the ground state.

In $CsGeI_3$, localization of excess electron forms metastable EP1* and EP2 configurations with formation energies of +0.45 eV and +0.25 eV, respectively. In EP1*, the electron is delocalized in the x-axis. It corresponds to a shallow state that lies 0.17 eV below CBM. Moreover, EP2 is located at 1.01 eV below CBM, exhibiting a 0.84 eV difference. Charge density and energy diagrams of metastable electron polaron configurations in $CsGeI_3$ are displayed in Figure 2.

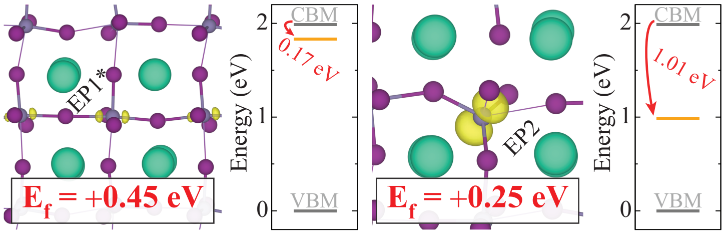

Figure 2: Isodensity surfaces (in yellow) of the single electron polaron configurations and corresponding formation energies for $CsGeI_3$. Isosurface levels are displayed at 0.005 eV/Å^3. Zero in the energy scale is set to the VBM.

In $CsGeBr_3$ and $CsGeI_3$, the double hole polaron forms a metastable state with the binding energy of +0.28 eV and +0.20 eV, respectively. The corresponding charge densities and energy diagrams are shown in Figure 3.

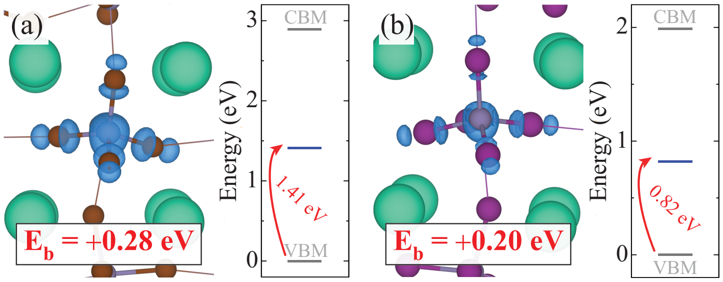

Figure 3: Isosurfaces of the metastable double hole polaron configurations and corresponding polaron formation energies in (a) $CsGeBr_3$ and (b) $CsGeI_3$. isosurface levels are displayed at 0.005 eV/Å^3. Zero in the energy scale is set to the VBM.

7 Metastable self-trapped exciton configurations

Beyond energetically favorable self-trapped exciton configurations that we report in the main text, our calculations reveal metastable self-trapped exciton configurations in $CsGeCl_3$ and $CsGeBr_3$, presented in Figure 4. In $CsGeCl_3$, the metastable STE involves coupling of an excess hole with EP1, localizing

on the same Ge atom with a binding energy of +0.20 eV. In this STE configuration, the single-particle level of the excess electron lies 2.56 eV below CBM, and the excess hole sits 1.09 eV above VBM. The difference between these single-particle levels is 0.45 eV.

In $CsGeBr_3$, the metastable STE forms by the excess hole coupling with EP2, where excess charge carriers localize on neighboring Ge sites. Here, the single-particle level for the excess electron is located 1.87 eV below CBM, meanwhile the single-particle level for the excess hole is located 1.03 eV above VBM. The difference between the electron and hole levels is 0.16 eV.

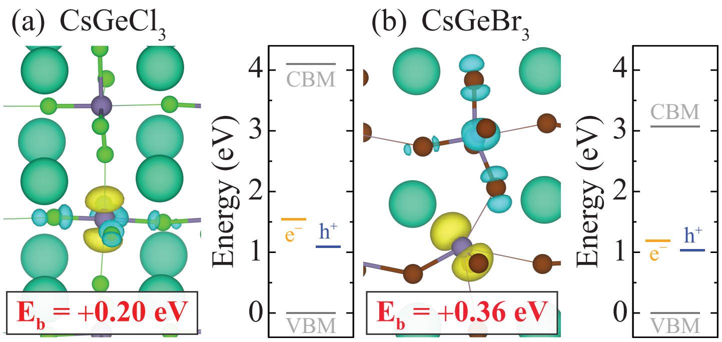

Figure 4: Isosurfaces of the metastable STE configurations and the corresponding energy diagram in (a) $CsGeCl_3$ and (b) $CsGeBr_3$. Subsets show the binding energy of STEs. The isosurface level is taken at 0.005 eV/Å^3. Zero in the energy scale is set to the VBM.

References

[1] Thomas Bischoff, Julia Wiktor, Wei Chen, and Alfredo Pasquarello. Nonempirical hybrid functionals for band gaps of inorganic metal-halide perovskites. *Physical Review Materials*, 3(12):123802, 2019.

[2] Christoph Freysoldt, Jörg Neugebauer, and Chris G Van de Walle. Fully ab initio finite-size corrections for charged-defect supercell calculations. *Physical Review Letters*, 102(1):016402, 2009.

[3] Stefano Falletta, Julia Wiktor, and Alfredo Pasquarello. Finite-size corrections of defect energy levels involving ionic polarization. *Physical Review B*, 102(4):041115, 2020. URL `https://doi.org/10.1103/PhysRevB.102.041115`.

[4] Herbert Fröhlich. Electrons in lattice fields. *Advances in Physics*, 3(11):325–361, 1954.

[5] Jozef T Devreese and Alexandre S Alexandrov. Fröhlich polaron and bipolaron: recent developments. *Reports on Progress in Physics*, 72(6):066501, 2009.

[6] Nguyen Thi Han, Vo Khuong Dien, and Ming-Fa Lin. Electronic and optical properties of $csgex_3$ (x= cl, br, and i) compounds. *ACS Omega*, 7(29):25210–25218, 2022.

[7] Thirumal Krishnamoorthy, Hong Ding, Chen Yan, Wei Lin Leong, Tom Baikie, Ziyi Zhang, Matthew Sherburne, Shuzhou Li, Mark Asta, Nripan Mathews, et al. Lead-free germanium iodide perovskite materials for photovoltaic applications. *Journal of Materials Chemistry A*, 3(47): 23829–23832, 2015.
