# Supplementary material for: Exploring the Polaron Landscape in Germanium Halide Perovskites: CsGeCl3, CsGeBr3, and CsGeI3
Source: J Phys Chem Lett. 2025 Dec 26;17(6):1569–75. doi: 10.1021/acs.jpclett.5c02516 (PMC12908147; doi:10.1021/acs.jpclett.5c02516)
Supplement: Supplementary file 2 [file jz5c02516_si_002.pdf]

jz-2025-02516r.R1

Name: Peer Review Information for "Exploring the Polaron Landscape in Germanium Halide Perovskites: CsGeCl₃, CsGeBr₃, and CsGeI₃"

First Round of Reviewer Comments

Reviewer: 1

Comments to the Author

Comments to the authors on:

Exploring the Polaron Landscape in Germanium Halide Perovskites:
CsGeCl3, CsGeBr3, and CsGeI3

The authors simulate ground state polaron properties for the interesting new material class Germanium halide perovskite CsGeX3 (X=Cl, Br, I). Their computational approach employs stateof-the-art DFT methods, including geometry optimizations with hybrid density functionals, polaron formation energy calculations with optimized Hartree-Fock exchange fractions, and finite-size corrections within supercell simulations. While this study represents a thorough computational investigation and appears to be the first polaron simulation work for these materials, several methodological limitations affect the scope of its impact and do not build forefront research. The results lack validation from higher-level theoretical methods, e.g., the GW method (charged excitations) for the polaron energy levels and their positions w.r.t. to the band edges and the BSE@GW method (neutral excitations) for computing the energy levels for the bound excitons. The position of the polaron energy levels w.r.t. to the band edges (e.g. important for optical applications) will strongly depend on the values of the band gap and the chosen density functional approximation. A comparison of hybrid DFT band gaps with GW band gaps would allow a theoretical validation of the results and an assessment of the quality of the chosen density function approximation. Clearly, these suggested additional calculations would be very expensive, but feasible and in lack of experimental results, this is what would be necessary to bring the work to the forefront.

The manuscript presents novel findings regarding these materials' properties and constitutes a valuable contribution for specialists in DFT polaron simulations, serving as an important foundation for future investigations in this material class. Based on these considerations, publication in an appropriate format of the Journal of Physical Chemistry would be suitable.

In addition, it would be important to extend the supplemental information with more details about the computed results for any form of publication. At the moment, only results are presented in the manuscript, but their meaning can't be validated because, e.g., the contributions from the finitesize corrections are not shown. They can make up a large portion of the corresponding quantities, so for experts, listing the individual contributions to the formation energies would be of high value. Here is a full list of things that are missing from my point of view:

- List the values of the finite-size corrections for each computed quantity
- Make the simulation data available, i.e. the final polaron and exciton geometries, all input and output files. At the moment, the results are not reproducible for external researchers.
- Validation of the Fulfillment of the Koopmans theorem for the polaron energy levels for the given fraction of HF exchange
- In connection with the previous point, quantities computed via Equation (1) will have strong dependency on the fraction of HF exchange. There are alternative ways to compute the formation energy with less dependency (see discussion e.g. in Sadigh, Erhardt, Årberg 2015 Phys. Rev. B 92 075202 and Kokott et al. 2018, New J. Phys. 20 033023). A discussion why the chosen approach is reliable would support the findings. Alternatively, it could be demonstrated
- In equation (1), the contribution E_0 is not introduced. Also, the finite-size correction terms are not included in equation (1).
- The band gaps of the materials are not listed (although they can be derived from Table 2).
- An analyses of the orbital character of the polarons would be interesting (e.q. showing projected density of states and/or projected Mulliken charge analysis).
- A more detailed discussion of why the space groups of the systems in the manuscript are different from previously reported space groups. How much different are they energetically?
- It would be important to mention (especially for non-expert readers) that an adiabatic approached is used to describes polaron formation. Polarons with small formation energy may however require non-adiabatic treatment and this may alter the formation even qualitatively. An adiabatic approximation is justifiable if strong electron-phonon coupling is dominating. The authors give no statement on the strength of the electron-phonon coupling in these materials (e.g. via Fröhlich coupling constant).

Reviewer: 2

Comments to the Author

The manuscript entitled " Exploring the Polaron Landscape in Germanium Halide

Perovskites: $CsGeCl_3$, $CsGeBr_3$, and $CsGeI_3$" needs major revision. The detail comments which need to be addressed are as follows:

1) The author mentioned that “The auxiliary density matrix method (ADMM) is employed in the calculations, which are run in the PBE0(α) hybrid functional level of theory. The α parameters, 0.32, 0.26, and 0.21 for $CsGeCl_3$, $CsGeBr_3$, $CsGeI_3$ respectively, are based on Ref.”.

Here, what is the physical significance of α? Why is it important to introduce with the hybrid functional such as PBE0?

2) What is the significance of the electrostatic finite-size correction term ‘E_{corr}’ to calculate the polaron formation energy, E_f of self-trapped states, as the term is not included in equation 1.

3) Is there any reason to carry out the analysis at 0 K considering the monoclinic structure. Because in practical, the same phenomenon will have to occur at room temperature or even higher.

Besides that, is there any effect of temperature on the formation of polaronic states?

4) The author mentioned that “We carry out geometry optimization with a high fraction of exact exchange (α = 0.50) to facilitate charge localization.” Moreover, they also mentioned that “Resulting systems that contain a localized electron are further optimized using the optimal fraction of the exact exchange ($CsGeCl_3$: 0.32, $CsGeBr_3$: 0.26, $CsGeI_3$: 0.21).”

Here, the author should provide proper justification for using optimal fraction values of exact exchange such as 0.32, 0.26, and 0.21 after considering a high fraction of the same, i.e., 0.50.

5) In the beginning the author mentioned that “Charge localization can significantly influence electronic and optical properties in perovskites. Localized charge carriers, such as polarons, have been reported to enhance the nonlinear optical response, or reduce charge carrier mobility”. However, the manuscript is lack of such perspectives. Therefore, it

is suggested to include how the formation of different polaronic states affects the optoelectronic properties of $CsGeX_3$ perovskites.

Author's Response to Peer Review Comments:

Dear Editor,

Thank you for considering our manuscript “Exploring the Polaron Landscape in Germanium Halide Perovskites: $CsGeCl_3$, $CsGeBr_3$, and $CsGeI_3$” for publication. We also thank the referee for their response and helpful suggestions. We have improved our manuscript based on their comments. We hereby submit our reply to these comments along with a revised version of our manuscript.

This letter contains a detailed response to the referees' comments (in blue) and an overview of changes to the manuscript. The resubmission also includes a marked-up version of the manuscript, in which changes to the manuscript have been highlighted. We hope that we have satisfactorily addressed all the comments by the referees and that our manuscript can be accepted for publication in The Journal of Physical Chemistry Letters.

With kind regards,
Mehmet Baskurt.

Report of the First Reviewer – jz-2025-02516r/Baskurt

Comments:

The authors simulate ground state polaron properties for the interesting new material class Germanium halide perovskite CsGeX3 (X=Cl, Br, I). Their computational approach employs state-of-the-art DFT methods, including geometry optimizations with hybrid density functionals, polaron formation energy calculations with optimized Hartree-Fock exchange fractions, and finite-size corrections within supercell simulations. While this study represents a thorough computational investigation and appears to be the first polaron simulation work for these materials, several methodological limitations affect the scope of its impact and do not build forefront research. The results lack validation from higher-level theoretical methods, e.g., the GW method (charged excitations) for the polaron energy levels and their positions w.r.t. to the band edges and the BSE@GW method (neutral excitations) for computing the energy levels for the bound excitons. The position of the polaron energy levels w.r.t. to the band edges (e.g. important for optical applications) will strongly depend on the values of the band gap and the chosen density functional approximation. A comparison of hybrid DFT band gaps with GW band gaps would allow a theoretical validation of the results and an assessment of the quality of the chosen density function approximation. Clearly, these suggested additional calculations would be very expensive, but feasible and in lack of experimental results, this is what would be necessary to bring the work to the forefront.

Our Reply:
We thank the reviewer for raising this important point regarding validation from higher-level theoretical methods such as GW and BSE@GW. In this work, we carried out calculations at PBE0(a) level where the fraction of exact exchange is determined by enforcing the generalized Koopmans’ condition. This approach has already been benchmarked by Bischoff *et al* (*Phys. Rev. Mater.* **2019**, 3, 123802) against quasiparticle self-consistent GW (QSGW) calculations across several halide perovskites, including germanium halide perovskites, and validated to yield band gaps with good agreement to that of QSGW. We emphasized the benchmarking done in the aforementioned reference (co-authored by one of us) in the revised manuscript.

The manuscript presents novel findings regarding these materials' properties and constitutes a valuable contribution for specialists in DFT polaron simulations, serving as an important foundation for future investigations in this material class. Based on these considerations, publication in an appropriate format of the Journal of Physical Chemistry would be suitable.

In addition, it would be important to extend the supplemental information with more details about the computed results for any form of publication. At the moment, only results are presented in the manuscript, but their meaning can’t be validated because, e.g., the contributions from the finite-size corrections are not shown. They can make up a large portion of the corresponding quantities, so for experts, listing the individual contributions to the formation energies would be of high value. Here is a full list of things that are missing from my point of view:

- List the values of the finite-size corrections for each computed quantity

Our Reply:
Following the reviewer’s request, values of the finite-size corrections are listed in the SI of the revised manuscript.

- Make the simulation data available, i.e. the final polaron and exciton geometries, all input and output files. At the moment, the results are not reproducible for external researchers.

Our Reply:
Simulation data consisting the final polaron and exciton geometries, input, and output files are made available in ZENODO: DOI: 10.5281/zenodo.17129842 . The dataset is not yet published, however the link is generated and files can be accessed: https://zenodo.org/records/17129842?preview=1&token=eyJhbGciOiJIUzUxMiJ9.eyJpZCI6IjA4YTU2OTAxLTVjZTQtNGFmYy04OGZhLTkyNjAzOWE5MjdkMCIsImRhdGEiOnt9LCJyYW5kb20iOiIxZmNlNTMwN2ViODllYTgyMzVkNGIzMjgxYjEwZjBlMiJ9.yn4MOcCg4B1C1Jouc1H_YBe6XF72lmY29Bhi-Ih5Ir6lNWH9W2U6alu19Vjz7pQndw4BrusOU-Bc4vTt1yuRUw

- Validation of the Fulfillment of the Koopmans theorem for the polaron energy levels for the given fraction of HF exchange

Our Reply:
Koopmans theorem for the polaron energy levels in $CsGeX_3$ perovskites are given in the SI of the revised manuscript. The changes are as follows:

In this work, we investigate polaron formation in $CsGeX_3$ perovskites using the PBE0(α) hybrid functional. The fractions of Hartree-Fock exchange (α) are taken from Ref. 1, where α was determined non-empirically by enforcing the generalized Koopmans’ condition for halogen vacancy states. To validate the applicability of these α values for localized carrier states in the present systems, we explicitly tested the Koopmans’ condition for the polaron energy levels, specifically for the electron polaron configurations EP1 and EP2. For EP1, we find the optimal value for the HF exchange fraction α to be 0.36, 0.32, and 0.29 for $CsGeCl_3$, $CsGeBr_3$, and $CsGeI_3$, respectively, after applying finite-size corrections to the single-particle levels (see Figure 1). For EP2, the corresponding values are 0.36, 0.30, and 0.27, respectively. These values are higher than those reported for vacancy states in Ref. 1 (0.32, 0.26, 0.21). However, Ref. 1 also found that other localized states, modeled using adjustable potential probes, yielded somewhat higher α values. This indicates that such variations are expected

and confirms that the previously reported α values provide a self-interaction-free description of localized charges, which we therefore adopt throughout the main calculations for consistency.

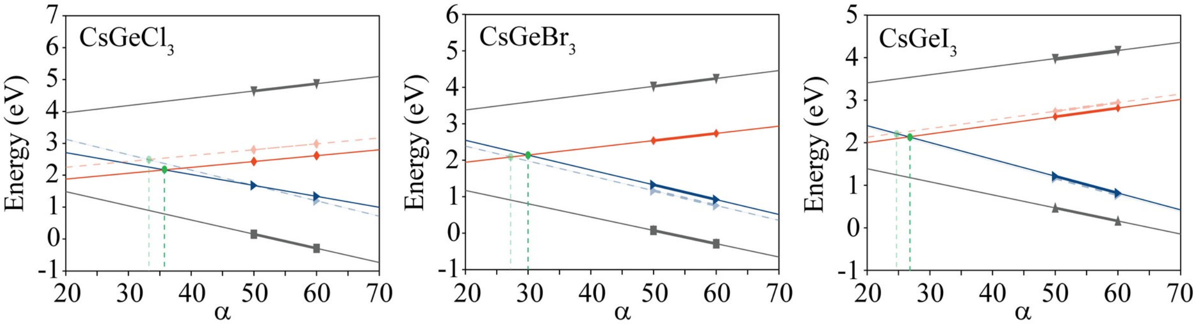

Figure 1: Band edges and single particle levels of electron polaron EP1 in $CsGeCl_3$, $CsGeBr_3$, and $CsGeI_3$. Blue and red lines represent the occupied and unoccupied single-particle levels, respectively. Single-particle levels with solid lines are corrected levels while dashed lines are the uncorrected levels. Green points indicate the HF fraction that satisfy Koopmans' condition.

- In connection with the previous point, quantities computed via Equation (1) will have strong dependency on the fraction of HF exchange. There are alternative ways to compute the formation energy with less dependency (see discussion e.g. in Sadigh, Erhart, Årberg 2015 Phys. Rev. B 92 075202 and Kokott et al. 2018, New J. Phys. 20 033023). A discussion why the chosen approach is reliable would support the findings. Alternatively, it could be demonstrated

Our Reply:
We thank the reviewer for highlighting important works which propose alternative frameworks to reduce the dependency on the fraction of HF exchange. As the reviewer points out, formation energies evaluated via Equation (1) has strong dependency on the fraction of HF exchange. However, in this work, we use the HF exchange fraction that satisfy the generalized Koopmans' condition. This procedure has been benchmarked against quasiparticle self-consistent GW in several halide perovskites (Bischoff et al, *Phys. Rev. Mater.* **2019**, 3, 123802). We acknowledge the alternative ways to compute the polaron formation energy and cited these relevant articles in the revised manuscript.

- In equation (1), the contribution E_0 is not introduced. Also, the finite-size correction terms are not included in equation (1).

Our Reply:
E_0 accounts for the finite-size correction where the static dielectric constant is effective, which is equal to the E_corr in the text. Equation 1 is updated in the revised manuscript to avoid conflicts.

- The band gaps of the materials are not listed (although they can be derived from Table 2).

Our Reply:
The band gaps of $CsGeX_3$ are listed in the SI of the revised manuscript.

- An analyses of the orbital character of the polarons would be interesting (e.q. showing projected density of states and/or projected Mulliken charge analysis).

Our Reply:
Information regarding the orbital character of the polarons from projected density of states are provided in the revised manuscript. The changes are as follows:

To identify the orbital character of the localized charge carriers, we computed spin-polarized atom-projected density of states for the Ge atom hosting the polaron in the supercell. For hole polarons, an in-gap state appears in the β (spin-down) channel (Fig S3 a and b), and for electron polarons, the in-gap state appears in the α (spin-up) channel (Fig S3 c,d, and e). In the electron polaron configurations, the in-gap state is dominated by Ge(*p*) character whereas the hole polaron level is primarily Ge(*s*). Moreover, single hole polaron shows non-negligible Ge(*p*) contribution. Because the same qualitative features are obtained for $CsGeCl_3$, $CsGeBr_3$, and $CsGeI_3$, we show $CsGeCl_3$ as a representative case.

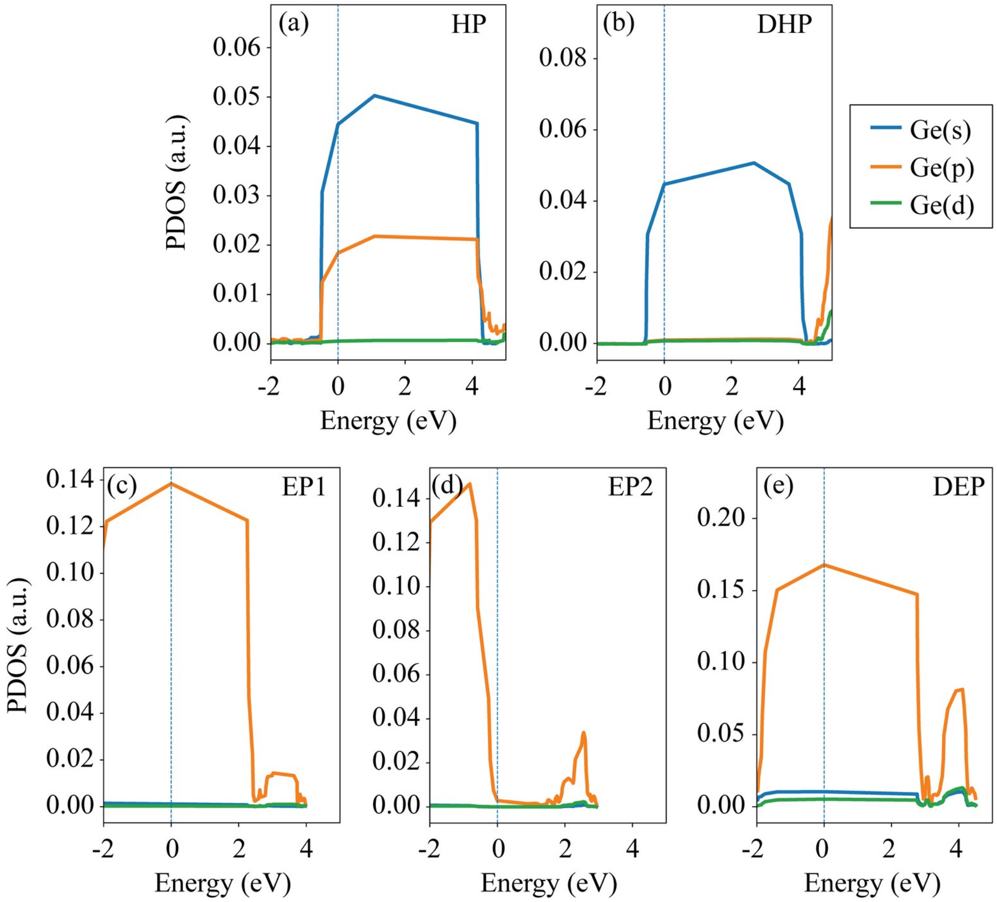

Figure S3: PDOS for the Ge atom hosting the localized hole(s) and electron(s).

- A more detailed discussion of why the space groups of the systems in the manuscript are different from previously reported space groups. How much different are they energetically?

Our Reply:
A more detailed energy difference between different space groups of $CsGeX_3$ systems are added in the SI of the revised manuscript.

- It would be important to mention (especially for non-expert readers) that an adiabatic approached is used to describes polaron formation. Polarons with small formation energy may

however require non-adiabatic treatment and this may alter the formation even qualitatively. An adiabatic approximation is justifiable if strong electron-phonon coupling is dominating. The authors give no statement on the strength of the electron-phonon coupling in these materials (e.g. via Fröhlich coupling constant).

Our Reply:
We thank the reviewer for pointing this out. We have clarified that our treatment of polaron formation is adiabatic in the revised manuscript. Additionally, we calculated the Fröhlich coupling constants, we reveal strong electron-phonon coupling in these materials, supporting the use of the adiabatic approximation is valid for $CsGeX_3$. The changes are as follows:

In the revised manuscript: "… We use an adiabatic (Born-Oppenheimer) description of polaron formation. For CsGeX_3 perovskites, Fröhlich constants show weak to intermediate coupling; therefore the non-adiabatic electron-phonon effects primarily renormalize transport and are not expected to alter the presence of self-trapped minima. Therefore, adiabatic descriptions for polaron formation in GHPs are justifiable (see SI for further analysis and discussion on electron-phonon coupling strength). "

In SI: "In this work, we evaluate polaron formation adiabatically by adding/removing electronic charge and relaxing the lattice on a single potential energy surface. Since polarons with small formation energies can be sensitive to non-adiabatic effects, we quantify the long-range electron-phonon coupling in GHPs via the Fröhlich constant, α,

$$\alpha = \frac{e^2}{4\pi\epsilon\hbar}\sqrt{\frac{m^*}{2\hbar\omega_{\text{LO}}}}(\frac{1}{\epsilon_\infty} - \frac{1}{\epsilon_0}),$$

where m* is the carrier effective mass, ω_{LO} is the polar LO phonon frequency at Γ, ϵ_∞ is the high-frequency dielectric tensor, and ϵ_0 is the static dielectric tensor. Values are given in Table S2.

	ω_{LO} (THz)	ϵ_∞ (x,y,z)	ϵ_0 (x,y,z)	m_e^*/m_e	m_h^*/m_e	α
$CsGeCl_3$	9.134	3.695, 3.695, 3.695	13.91, 13.91, 12.13	0.272	0.284	2.54
$CsGeBr_3$	6.20	6.55, 6.55, 6.55	11.07, 11.07, 11.07	0.176	0.181	0.63
$CsGeI_3$	5.053	6.215, 6.215, 6.215	20.96, 20.96, 20.96	0.137	0.143	1.275

Table S2: LO phonon frequency at Γ (ω_{LO}), directional components of high-frequency (ϵ_∞) and static (ϵ_0) dielectric tensors, electron (m_e^*/m_e) and hole (m_h^*/m_e) effective masses,[4,5] and resulting Fröhlich constant α.

The Fröhlich constants show weak to intermediate coupling strength across the series, with 2.54 ($CsGeCl_3$) > 1.28 ($CsGeI_3$) > 0.63 ($CsGeBr_3$). These values point to intermediate-coupling behavior rather than the strong small-polaron limit. In this α range, static relaxations dominate the stabilization energy while non-adiabatic corrections primarily renormalize transport and are not expected to alter the presence of self-trapped minima, therefore not alter polaron formation qualitatively."

Report of the Second Reviewer – jz-2025-02516r/Baskurt

Comments:

The manuscript entitled "Exploring the Polaron Landscape in Germanium Halide Perovskites: $CsGeCl_3$, $CsGeBr_3$, and $CsGeI_3$" needs major revision. The detail comments which need to be addressed are as follows:

1) The author mentioned that "The auxiliary density matrix method (ADMM) is employed in the calculations, which are run in the PBE0(α) hybrid functional level of theory. The α parameters, 0.32, 0.26, and 0.21 for $CsGeCl_3$, $CsGeBr_3$, $CsGeI_3$ respectively, are based on Ref.".
Here, what is the physical significance of α? Why is it important to introduce with the hybrid functional such as PBE0?

Our Reply:
Semilocal functionals like PBE experience problems describing the electronic structure of semiconductors. They tend to underestimate the band gap and delocalize charges of a system drastically due to the self-interaction error, where an electron interacts with itself through the Coulomb (Hartree) term. Therefore, they cannot properly describe localized states. This problem can be treated by using a hybrid functional such as PBE0(α), where the exchange-correlation energy is calculated as a mix of DFT exchange and Hartree Fock (HF) exchange:

$$E_{xc}^{PBE0(\alpha)} = \alpha E_x^{HF} + (1-\alpha)E_x^{PBE} + E_c^{PBE} \ .$$

Here, the α parameter controls the fraction of HF exact exchange is mixed in this hybrid functional. For systems where polaron formation and self-trapped exciton binding are central, the choice of α strongly effects the stability and localization of the polaronic states. The fraction of the HF exchange can be derived by enforcing the Koopmans' condition. This ensures that the single-particle levels are constant and do not depend on their occupation. At the fraction α that satisfy Koopmans' condition, the accuracy of the system is improved by minimizing the self-interaction error. In this work, we adopt α values derived by enforcing generalized Koopmans' condition, from the literature (Bischoff *et al. Phys. Rev. Mater.* **2019**, 3, 123802).

In the revised manuscript we included an explanation why hybrid functional such as PBE0 is important and what significance α has. The regarding changes are as follows:
"We carry out geometry optimization with a high fraction of exact exchange (α = 0.50) to facilitate charge localization. Here, using a high fraction of exact exchange, we bias the system towards charge localization. This step stabilizes a localized state and lets the lattice to relax into the polaronic configuration. We identify two distinct single electron polaron configurations, which we call EP1 and EP2. The EP1 is a more symmetric structure while the EP2 configuration corresponds to an enhanced off-centering of Ge. We then mimic the initial distortions to form EP1 and EP2 states in CsGeBr3 and CsGeI3 and optimize the structure with the PBE0(0.50) functional. Resulting systems that contain a localized electron are further optimized with the physically justified, optimal fraction of the exact exchange (CsGeCl3 : 0.32, CsGeBr3 : 0.26, CsGeI3 : 0.21). This two-step procedure ensures that the final polaron properties are evaluated avoding convergence of artificially delocalized solutions."

2) What is the significance of the electrostatic finite-size correction term 'E_{corr}' to calculate the polaron formation energy, E_f of self-trapped states, as the term is not included in equation 1.

Our Reply:

In charged supercells, electrostatic interactions between periodic images of the localized charge can affect the polaron formation energy. If this is not corrected, the calculated formation energy will be size-dependent. In the revised manuscript, we revise equation 1, and list the values of finite-size corrections in the SI.

3) Is there any reason to carry out the analysis at 0 K considering the monoclinic structure. Because in practical, the same phenomenon will have to occur at room temperature or even higher. Besides that, is there any effect of temperature on the formation of polaronic states?

Our Reply:
Our polaron formation energies are obtained from static 0K relaxations, considering the monoclinic ground state structures. This approach isolates the intrinsic energetics of the localized states without any thermal fluctuations. While the stability of polarons can be influenced by finite-temperature effects, addressing these would require computationally expensive hybrid DFT AIMD simulations in large supercells. Therefore, here we focus on the 0K energetics as a well defined reference.

4) The author mentioned that "We carry out geometry optimization with a high fraction of exact exchange ($\alpha = 0.50$) to facilitate charge localization." Moreover, they also mentioned that "Resulting systems that contain a localized electron are further optimized using the optimal fraction of the exact exchange ($CsGeCl_3$: 0.32, $CsGeBr_3$: 0.26, $CsGeI_3$: 0.21)."
Here, the author should provide proper justification for using optimal fraction values of exact exchange such as 0.32, 0.26, and 0.21 after considering a high fraction of the same, i.e., 0.50.

Our Reply:

Geometry optimization of the structures with initial local distortions may remain delocalized across the supercell since semilocal screening favors delocalization. Using a high fraction of exact exchange, we bias the system towards charge localization. This stabilizes a localized state and lets the lattice to relax into the polaronic configuration. The resulting localized states are then reoptimized with the physically justified, Koopmans-tuned α values ($CsGeCl_3$: 0.32, $CsGeBr_3$: 0.26, $CsGeI_3$: 0.21). This two-step procedure ensures that the final polaron properties are evaluated avoiding convergence to artificially delocalized solutions.

In order to improve the clarity, we expanded the methodology in the revised manuscript.

5) In the beginning the author mentioned that "Charge localization can significantly influence electronic and optical properties in perovskites. Localized charge carriers, such as polarons, have been reported to enhance the nonlinear optical response, or reduce charge carrier mobility". However, the manuscript is lack of such perspectives. Therefore, it is suggested to include how the formation of different polaronic states affects the optoelectronic properties of $CsGeX_3$ perovskites.

Our Reply:
We thank the referee for pointing this out. Following their request, we have included a brief discussion in the revised manuscript.

jz-2025-02516r.R2

Name: Peer Review Information for "Exploring the Polaron Landscape in Germanium Halide Perovskites: CsGeCl₃, CsGeBr₃, and CsGeI₃"

Second Round of Reviewer Comments

Reviewer: 1

Comments to the Author

Comments to the authors on:
Exploring the Polaron Landscape in Germanium Halide Perovskites:
CsGeCl3, CsGeBr3, and CsGeI3

Thank you to the authors for addressing all of my concerns. I have only a few questions left with respect to the revised manuscript and supplemental information (SI):

(1) I think the following statement in the manuscript requires references: “Stable formation of different polaronic states in CsGeX3 perovskites can reduce charge transport through carrier localization. Moreover, strong local electron-phonon coupling in these materials can enhance polarizability and third-order susceptibility, which can enhance optical nonlinearity. “

(2) In the reply from the authors, the authors state: “Additionally, we calculated the Fröhlich coupling constants, we reveal **strong electron-phonon coupling** in these materials, supporting the use of the adiabatic approximation is valid for CsGeX3”. However, in the manuscript the authors write: “For CsGeX_3 perovskites, Fröhlich constants show **weak to intermediate coupling**; therefore the non-adiabatic electron-phonon effects primarily renormalize transport and are not expected to alter the presence of self-trapped minima.” This is contradicting and needs clarification.

(3) Also the statement: “Fröhlich constants show weak to intermediate coupling; therefore the non-adiabatic electron-phonon effects primarily renormalize transport and are not expected to alter the presence of self-trapped minima.” requires a reference.

(4) The finite size corrections for the double polarons are not listed in the SI.

(5) Section 1 in SI is not referenced in the manuscript as far as I can see. I think this point is most important to underline that the fraction of Hartree-Fock exchange has been validated and, thus, the method provides probably good quantitative results.

Reviewer: 2

Comments to the Author

After reviewing the authors' responses and the corresponding revisions made to the manuscript, it is clear that all major concerns have been adequately addressed. The revisions have significantly improved the quality and clarity of the work. Therefore, the authors' responses can be considered as satisfactory, and the manuscript is recommended for publication in its current form.

Author's Response to Peer Review Comments:

Dear Editor,

Thank you for considering our revised manuscript titled "Exploring the Polaron Landscape in Germanium Halide Perovskites: $CsGeCl_3$, $CsGeBr_3$, and $CsGeI_3$". We are grateful to the reviewers' careful assessments. We hereby submit our reply to the reviewers' comments along with a revised version of our manuscript.

This letter contains a point-by-point response (in blue) to the reviewer comments and an overview of changes to the manuscript. In addition, the resubmission includes a marked-up manuscript, in which changes to the manuscript have been highlighted. We hope that all the comments have been satisfactorily addressed and that our manuscript can be accepted for publication in The Journal of Physical Chemistry Letters.

Kind regards,
Mehmet
Baskurt.

Report of the First Reviewer – jz-2025-02516r.R1/Baskurt

Comments:

Thank you to the authors for addressing all of my concerns. I have only a few questions left with respect to the revised manuscript and supplemental information (SI):

(1) I think the following statement in the manuscript requires references: "Stable formation of different polaronic states in CsGeX3 perovskites can reduce charge transport through carrier localization. Moreover, strong local electron-phonon coupling in these materials can enhance polarizability and third-order susceptibility, which can enhance optical nonlinearity. "

Our Reply:

We thank the reviewer for the request for references for this statement. This statement was given as a perspective based on similar effects in other materials, which are referenced in the introduction. However, direct reports on transport or third-order susceptibility changes by localized charges in germanium halide perovskites are lacking in the literature, to the best of our knowledge. To avoid overgeneralization, we reworded the text as expectation by analogy to related systems, and indicated that quantitative transport and optical nonlinearity analysis is outside of the present work in the revised manuscript.

The updated part is given below:

"Given the localized states we compute, such as single/double polarons and STEs, charge transport is expected to be reduced through carrier localization, and local electron-phonon coupling may contribute to enhanced polarizability and third-order susceptibility, as for example observed in Ref. 29-32,34, which can enhance optical nonlinearity. "

(2) In the reply from the authors, the authors state: "Additionally, we calculated the Fröhlich coupling constants, we reveal strong electron-phonon coupling in these materials, supporting the use of the adiabatic approximation is valid for CsGeX3". However, in the manuscript the authors write: "For CsGeX_3 perovskites, Fröhlich constants show weak to intermediate coupling; therefore the nonadiabatic electron-phonon effects primarily renormalize transport and are not expected to alter the presence of self-trapped minima." This is contradicting and needs clarification.

Our Reply:

We thank the reviewer for catching the inconsistency in our statement. In our previous response, 'strong electron-phonon coupling' was a wording error. As stated in the manuscript, our calculated Fröhlich constants are weak to intermediate coupling, ranging from 0.65 to 2.54. Importantly, this does not affect the conclusion: the Fröhlich coupling constant describes long-range polaron interactions (large-polaron behavior), while the stability of localized polaronic and excitonic states observed in our first-principles calculations arises from additional short-range and anharmonic contributions. These two aspects are complementary and not contradictory. Given that the computed polaron formation energies (0.2-1.5 eV) are much larger than the typical phonon energies (tens of meV), the adiabatic approximation remains appropriate for describing

the energetics of the self-trapped states. Nonadiabatic effects are expected mainly to influence carrier transport rather than the existence of localized minima. We have revised both the main text and the SI to clarify these points and ensure consistent terminology.

(3) Also the statement: “Fröhlich constants show weak to intermediate coupling; therefore the non-adiabatic electron-phonon effects primarily renormalize transport and are not expected to alter the presence of self-trapped minima.” requires a reference.

Our Reply:

We thank the reviewer for pointing the missing references. Related works are referenced in the revised manuscript.

(4) The finite size corrections for the double polarons are not listed in the SI.

Our Reply:

Finite-size corrections for the double polarons are listed in the SI of the revised manuscript. The updated table is given below:

	Single polaron			Double polaron		
	q	E_{corr} (eV)	ϵ_{corr} (eV)	q (eV)	E_{corr} (eV)	ϵ_{corr}
$CsGeCl_3$	$+$	$+0.07$	-0.14	$+2$	$+0.28$	-0.28
	$-$	$+0.07$	$+0.14$	-2	$+0.28$	$+0.28$
$CsGeBr_3$	$+$	$+0.08$	-0.16	$+2$	$+0.32$	-0.32
	$-$	$+0.08$	$+0.16$	-2	$+0.32$	$+0.32$
$CsGeI_3$	$+$	$+0.04$	-0.07	$+2$	$+0.15$	-0.15
	$-$	$+0.04$	$+0.07$	-2	$+0.15$	$+0.15$

(5) Section 1 in SI is not referenced in the manuscript as far as I can see. I think this point is most important to underline that the fraction of Hartree-Fock exchange has been validated and, thus, the method provides probably good quantitative results.

Our Reply:

We thank the reviewer for careful reading. We referenced the section 1 in SI in the revised manuscript. The part from the revised manuscript:

“… We validate the applicability of these adapted α values for localized carrier states in the present systems by explicitly testing the Koopmans’ condition for the polaron energy levels (see section in SI).”

Additionally, we updated the supporting information description as follows:

“Validation of Hartree-Fock exchange fraction in PBE0(α), finite-size corrections, structural properties of CsGeX3, Adiabatic treatment of polaron formation in GHPs, orbital character of the

polarons, metastable polaron configurations, metastable self-trapped exciton configurations. (pdf)”

Report of the Second Reviewer – jz-2025-02516r.R1/Baskurt

Comments:

After reviewing the authors’ responses and the corresponding revisions made to the manuscript, it is clear that all major concerns have been adequately addressed. The revisions have significantly improved the quality and clarity of the work. Therefore, the authors’ responses can be considered as satisfactory, and the manuscript is recommended for publication in its current form.

Our Reply:

We thank the reviewer for their positive evaluation and recommendation for publication.
